# Patients' experiences and attitudes toward the use of real‐time artificial intelligence‐assisted colonoscopy

**DOI:** 10.1111/codi.70574

**Published:** 2026-08-07

**Authors:** Ronja M. B. Lagström, Marianne Krogsgaard, Astrid Kindt, Bahara Besmalahi, Mustafa Bulut

**Affiliations:** ^1^ Department of Surgery Zealand University Hospital Køge Denmark; ^2^ Department of People and Technology Roskilde University Roskilde Denmark; ^3^ Department of Clinical Medicine University of Copenhagen Copenhagen Denmark; ^4^ Copenhagen Academy for Medical Education and Simulation (CAMES) Copenhagen Denmark

**Keywords:** artificial intelligence, colonoscopy, computer‐assisted diagnosis, patient acceptance of health care, qualitative research

## Abstract

**Background:**

There is limited knowledge about how patients experience artificial intelligence (AI) in healthcare. Concerns regarding safety and quality could be a barrier between patients and the healthcare system when implementing AI. It is crucial to understand patients' attitudes, and how they may react to AI being implemented when aiming to preserve their trust. We investigated patients' attitudes toward AI‐assisted colonoscopy to gain insights that can be used when introducing this new technology to future patients.

**Method:**

This is a qualitative study based on semi‐structured focus group interviews. Habile, Danish‐speaking adults who had received an AI‐assisted colonoscopy were invited. We conducted six focus group interviews, with 20 participants in total. All interviews were recorded and transcribed. Data were analysed using thematic analysis.

**Results:**

Patients described a sense of vulnerability in relation to undergoing a colonoscopy. Three main themes were identified. The first theme is ‘Empathy and professionalism—trust in AI‐assistance in colonoscopy depends on human factors’. The second and the third themes are ‘Information about AI should be proportional to the consequences’, and ‘Trying to make sense of AI during colonoscopy—balancing curiosity, irrelevance, and vulnerability’.

**Conclusion:**

Trust in AI‐assistance is dependent on trust in the clinicians. While AI has many strengths, it cannot provide empathy, which is essential for patients in distressing moments, such as during a colonoscopy. AI should be considered a supportive tool, while the endoscopist remains responsible for clinical decisions and patient outcomes. If written information about AI is provided, it should be informative but concise, avoiding details that generate more confusion than clarity.

**What does this paper add to the literature?**

During colonoscopy, patients can observe AI‐assisted detection and characterization in real‐time. Understanding patients' attitudes and how they may respond to the implementation of AI in colonoscopy is essential for maintaining their trust and ensuring compliance during the adoption of this new technology.

## BACKGROUND

AI‐assisted colonoscopy can improve the adenoma detection rate, potentially reducing the incidence of colorectal cancer (CRC) [1, 2]. The use of AI‐assistance does not increase the risk of procedure‐related complications, supporting its safe use during colonoscopy [2, 3]. The AI system is being increasingly recognized as a valuable diagnostic tool by clinicians [4], but the knowledge about patients' attitudes against AI‐assisted colonoscopy is limited [5].

Patients from different specialties generally hold positive views on the integration of AI in healthcare [6]. Previous experiences with illness, healthcare systems and information technologies, as well as socioeconomic factors, influence patients' perceptions about AI‐assistance [7].

Colonoscopy is a common procedure, aiding both diagnosis and treatment. Some patients are referred due to cancer suspicions, some as part of the CRC screening or post‐polypectomy surveillance programs, while others may be referred for suspected benign conditions such as inflammatory bowel disease. During an AI‐assisted colonoscopy, patients can view the detected polyps on the screen in real‐time. This immediate visibility sets AI‐assisted colonoscopy apart from other AI applications in healthcare, where results are typically reviewed by the clinician before being shared with the patient. This difference is crucial when determining whether patients should be informed about AI‐assistance during the procedure [8].

To maintain patients' trust in healthcare, it is essential to understand their attitudes and beliefs, and how they may respond to the implementation of the technology [7, 9, 10]. This also applies to colonoscopy, where the patient experience constitutes one of the key quality performance measures [11].

The aim of this study is to explore patients' attitudes toward AI‐assisted colonoscopy and to provide guidance for clinicians on introducing this technology, considering its potential impact on patient trust, and preferences for being informed of its use. Understanding these factors is essential for ensuring patient compliance with AI‐assisted colonoscopy.

## METHODS

### Study design

We conducted a qualitative study based on semi‐structured focus group interviews. The study is reported according to the Consolidated Criteria for Reporting Qualitative Research (COREQ) checklist [12].

### Setting and participants

All interviews were arranged at Zealand University Hospital (ZUH) Køge, Denmark, where approximately 14,000 endoscopic procedures are performed every year. The patient population is highly diverse, as the unit provides colonoscopies to patients from the entire region. The AI system GI Genius™ (Medtronic, Dublin, Ireland) is being used as an adjunct to colonoscopy, processing colonoscopy images in real‐time. Detected mucosal lesions are simultaneously marked on the screen throughout the colonoscopy. Patients can observe the screen during the procedure.

**FIGURE 1:**
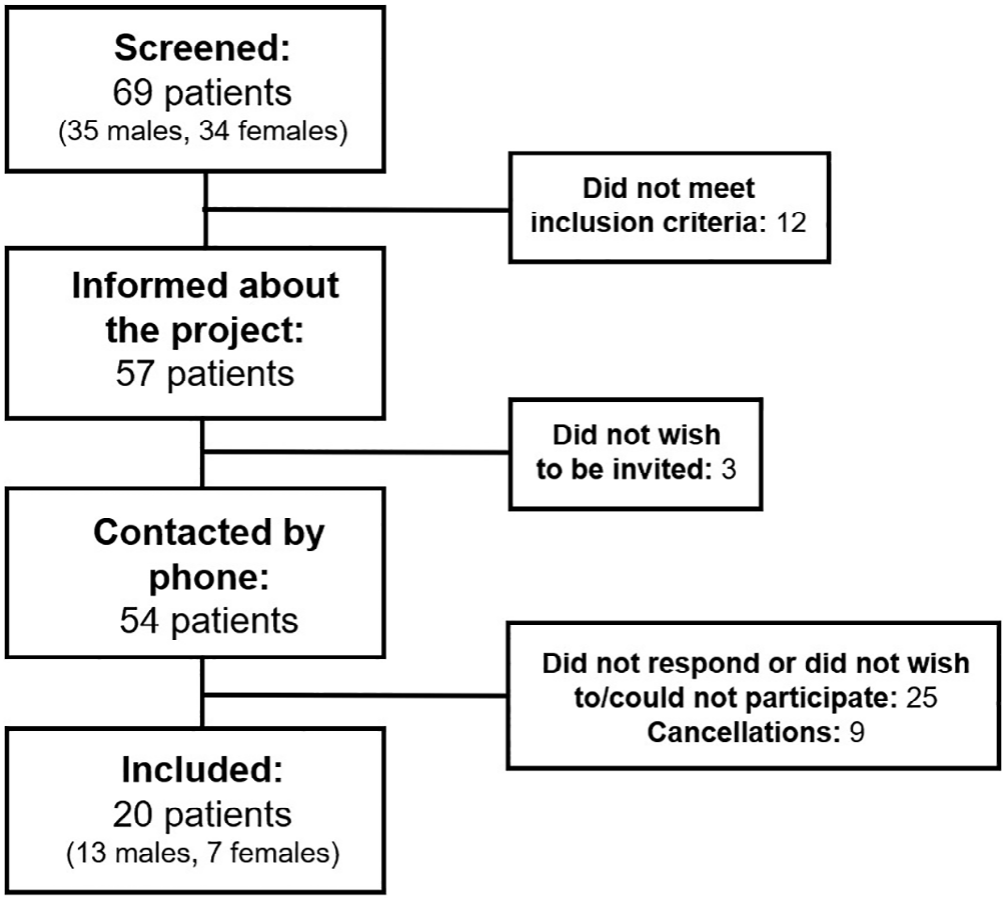
Flowchart of patient enrolment.

Patients 18 years of age or older who had received an AI‐assisted colonoscopy were invited. Moderate sedation with IV midazolam and fentanyl had been offered to all patients. Patients had to be habile and speak sufficient Danish. Patients with suspected cancer found during the colonoscopy were not included, since this AI system is designed solely to detect and characterize polyps. All patients received oral information about AI, either right before or during the procedure. This information was not standardized but adapted to the individual patient. All patients interested in participating in the study were provided with standardized written information after the procedure. Purposive sampling was used to ensure variation in the study population [13]. We aimed to include patients of different ages, sexes and socioeconomic statuses. Six focus group interviews with a total of 20 patients were conducted (Figure 1). Patient characteristics are shown in Table 1. Each focus group consisted of two to five participants (Table 2). Recruitment stopped when data saturation was reached [13].

**TABLE 1:** Patient characteristics.

	*N* (%)
Gender	
Male	13 (65%)
Female	7 (35%)
Age (years), mean (range)	62,25 (77–33)
Co‐habitants/married	19 (95%)
Employment status	
Working	13 (65%)
Retired	6 (30%)
Unemployed	1 (5%)
Level of education	
Primary or Lower Secondary Education	2 (10%)
Vocational Education	9 (45%)
Short‐ and Medium‐Cycle Higher Education	6 (30%)
Long‐Cycle Higher Education	3 (15%)
Colonoscopies received	
This was the first one	4 (20%)
Previously received one or more	16 (80%)
Reason for colonoscopy	
CRC‐screening with FIT	5 (25%)
Post‐polypectomy surveillance	4 (20%)
Referral on suspicion of cancer	9 (45%)
Diagnostic colonoscopy	1 (5%)
Control after surgery for CRC	1 (5%)
Moderate sedation (IV midazolam) and analgesics (IV fentanyl)	14 (70%)
Time passed between colonoscopy and interview (days), mean (range)	28,1 (4–111)

**TABLE 2:** Number of participants in interviews (*n* = 20).

Group number	Male/female	Character
1	4/1	A, B, C, D, E
2	3/1	F, G, H, I
3	2/0	J, K
4	0/2	L, M
5	4/0	N, O, P, Q
6	0/3	R, S, T

### Patient and public involvement (PPI)

Three patients in the endoscopy unit reviewed the written patient information before the interviews. Their feedback was used to ensure clear and sufficient information. In this way, patient and public involvement (PPI) ensured that patients received the information needed to make a voluntary and informed decision about study participation [14, 15]. We used an involvement matrix when planning the PPI [16].

### Data collection

Focus group interviews were chosen because they are suitable to provide insights into the sources of behaviours and motivations and to explore the range of opinions and experiences [17]. An interview guide was created prior to the first interview and used for all interviews. The interviews were semi‐structured and funnel‐based, with main questions and probes, starting with open questions and ending with more structured ones [18, 19]. All interviews were conducted by the first author (MD, PhD student) and moderated by a co‐author. The interviews were conducted in private rooms at the hospital. They were recorded (average lengths 85 min) and transcribed verbatim (in total 288 pages).

### Data analysis and interpretation

All transcripts were coded by the first author (RL) and validated by one experienced qualitative investigator (MK). Data were analysed by both authors (RL, MK) using thematic analysis [20, 21].

### Ethical considerations

Participating in the study was voluntary. All patients could withdraw their consent at any time. The primary investigator was responsible for the recruitment and talked to all patients prior to the procedure. All interested patients received written information about the study and were subsequently contacted by phone and invited to participate in the interview. Written consent was obtained on the day of the interview.

### Approval

According to the Danish Health Care Act § 46, subsection 5, this study was approved by the Head of the Department at Zealand University Hospital. It is registered at ‘The Region Zealand record of processing activities related to scientific research projects’ (p‐2024‐16974). The protocol is registered (28.10.2024) at osf.io/rvxfc/.

## RESULTS

The thematic analysis resulted in three themes: [1] Empathy and professionalism—trust in AI‐assistance in colonoscopy depends on human factors; [2] Information about AI should be proportional to the consequences; and [3] Trying to make sense of AI during colonoscopy—balancing curiosity, irrelevance, and vulnerability. An example of the thematic analysis is illustrated in Table 3.

**TABLE 3:** Example of thematic analysis.

A part of the structural thematic analysis: Theme 3 trying to make sense of AI during colonoscopy—Balancing curiosity, irrelevance and vulnerability		
Citations (what is said)	Meaning (what the text speaks about)	Theme
I don't think it's healthy for patients to lie there looking at that *[ed, endoscopic findings in the colon, marked by AI on the screen]*. I don't believe it's good. I don't think it is. It's irrelevant, because we don't understand it (I2). You get curious. And worried. And you want to know what it *[ed, endoscopic findings in the colon]* is (K3). I think it's interesting, the question of whether patients should be able to watch along or not. Because that's where AI makes a difference by giving a signal. Without AI, you wouldn't have a signal on the screen—you'd just watch along and think, well, the doctor is doing something there. But when you get that blinking signal, then of course suddenly there's a difference with AI (F2). It's such a sensitive topic that you need to mentally prepare yourself for bad news. And it can help if you've been following along a bit on the screen (K3). If you suspect that there's something going on, you might not want to do it *[ed, undergo a colonoscopy]*. But I went into it because I was completely sure that there was nothing. So, for me, I thought that there couldn't be any harm in looking (S6).	Patients are curious about their body and potential conditions. This curiosity can be both positive and anxious. Some wish to be in as much control as possible during the procedure, while others wish to get the procedure over with before they receive any kind of information about findings. There are concerns about the potential negative effects of looking at the screen during the examination of the bowel, where AI adds an extra layer of information. Some patients were rather neutral.	Trying to make sense of AI during colonoscopy – balancing curiosity, irrelevance, and vulnerability

### Theme 1: Empathy and professionalism—Trust in AI‐assistance in colonoscopy depends on human factors

Participants emphasized that human factors provided by clinicians were the most important element during a colonoscopy. Their trust in both the procedure and the use of AI depended on these interpersonal aspects, such as emotional intelligence and communication. Undergoing colonoscopy may evoke concerns related to discomfort during the procedure, its outcomes, as well as feelings of exposure and vulnerability: “*What weighed on me was the fear of what would be found. It was the human being that made it possible for me to be calm. The AI system didn't come over, put its arm around me and said, “it's going to be okay” (M4)*”. The patients' trust in clinicians was not affected by the involvement of AI.

Participants discussed their concerns regarding data leakage and misuse or programming errors leading to incorrect diagnoses or overlooked pathology. They were concerned that excessive reliance on the system by the endoscopist could lead to loss of knowledge and skills: ‘*The competence of the clinician performing the task will deteriorate over time. There is a potential loss of knowledge when we assign that task to machines. Then the power goes out, and suddenly we find ourselves in a society that has lost its skills’ (F2)*. Insufficient understanding of AI limitations by clinicians and the possibility of avoiding responsibility with potential consequences for the patient was also mentioned.

Participants generally agreed that AI surpasses humans in many aspects, providing a form of additional reassurance, but that AI should not make the decisions: ‘*I don't think it should do much more than simply inform. But it would be able to identify connections that might be difficult to see, because as a doctor, you can't possibly go through and process a thousand reports’ (G2)*. Human interaction and the abilities that clinicians possess are highly valued and desired by patients. Patients argued that some tasks cannot be taken over by a machine, particularly those involving interactive communication and the ability to treat patients within a broader clinical and personal context: ‘*The treatment of a human being on an individual level must never be decided by a machine. It's data and data processing. It doesn't possess empathy or ethical judgment’ (J3)*.

Some participants emphasized their confidence in AI and its ability to analyse large amounts of data, viewing AI as an independent and reliable entity: ‘Personally, *I'm probably at a point where I have more trust in AI. Once it has had that knowledge fed into it, I would consider it better at interpreting all the incoming data than a human brain is capable of. It gives me a sense of reassurance to have AI involved’ (J3)*.

### Theme 2: Information about AI should be proportional to the consequences

Some participants would have preferred receiving written information about the use of AI‐assistance prior to the procedure. This information may facilitate greater patient involvement, allowing them to follow the procedure. Furthermore, it would allow patients to opt out if they did not wish to undergo an AI‐assisted procedure, supporting patient autonomy: ‘*I would have liked to see it included in the invitation, stating there may be a possibility that you will be taken into this room where there's no turning back’ (J3)*. However, many participants did not believe patients should be given the option to choose whether AI was used, as it was viewed as part of a standard, voluntary procedure.

Other participants considered written information unnecessary, as AI was viewed as just one of many systems and tools used during the procedure. Excessive information might negatively affect patients during the examination: ‘*The information doesn't matter to me. Whether you use AI or not. For me, it just creates more questions. If you want to inform patients that AI is being used, you risk creating more questions. Are two tubes being used? Is it twice as big? And does it hurt four times as much?’ (I2)*.

Participants suggested that *if* information is given, it must be short and concise not to create additional uncertainties with unnecessary worrying as a result. Information should only contain a short description of the AI system, and what it is used for: ‘*Just a brief explanation about AI being used. That can be explained in five lines’ (N5)*.

Participants expressed concerns for the older patients, noting that the oldest segment of the population may be insufficiently prepared to adapt to technological developments in the healthcare sector. Participants suggested that we use the term ‘*a machine with pattern recognition’ (T6)* instead of AI to make it sound less frightening for the older generation.

### Theme 3: Trying to make sense of AI during colonoscopy—Balancing curiosity, irrelevance and vulnerability

Participants explained that, on one hand, seeing AI on the screen could be exciting, but on the other hand, they felt that the information was irrelevant because they were presented with data they could not interpret. It was mentioned that the potential consequences, specifically changes in diagnostic outcomes and treatment decisions, may influence whether patients should be able to view the screen, depending on the extent to which AI affects clinical decision‐making and subsequent follow‐up and treatment: ‘*The other thing I think will make a difference is whether AI affects whether something [ed, polyp/tissue] is removed, whether there is overtreatment, or how much is detected. Those aspects make a difference for me as a patient’ (F2)*. Some expressed being indifferent to the use of AI, emphasizing on the quality of treatment and not the specific equipment: ‘*Well, to me, this is completely uninteresting. It's a tool. I don't ask who made the knife the surgeon uses to cut, and so on. In my view, it's up to each hospital and each clinician to choose the best method, the best tool to do the best possible work — and AI is just a tool’ (C1)*.

Participants also discussed the concern that AI makes it more accessible for patients to take part in the diagnostic process, despite not being trained to do so. This was considered to potentially place an emotional burden on patients since unfiltered information presented on the screen may be overwhelming before being interpreted and translated to layman's terms by a clinician: ‘*I think that one should consider, from an ethical perspective, whether the patient should be sitting here and diagnosing. Because that is what they are being allowed to do’ (N5)*.

However, closely following the procedure and observing the AI output on the screen was also considered an advantage, as it could help preparing for the possibility of an unpleasant finding: ‘*I would like to know more about what the different indicators on the screen mean. That might mean I don't ask too many questions but can follow along. It also makes it easier for me to receive unpleasant news afterward, because I've been thinking about it beforehand’ (K3)*. Participants suggested establishing clear expectations between patients and clinicians regarding whether patients wish to be informed during the procedure. When discussing reactions to AI appearing on the screen, one participant reflected on their mixed feelings after the experience. Although it may be perceived as exciting, discomfort and anxiety associated with the procedure may leave patients with little capacity to engage with the use of AI: ‘*I regretted a bit afterwards that I didn't look. I thought it could have been fun to just see what it was — what it looked like — but on the other hand I also felt a bit uneasy about the situation, because I'd had an experience where it was very uncomfortable and very painful and so on’ (F2)*.

Other patients reported that they had not particularly noticed AI under the procedure or remembered being informed about it. Neither did they remember that AI was discussed between the clinicians in the room; they only remembered the things that were said directly to them. The use of AI was essentially invisible to many patients unless explicitly highlighted. ‘*And my experience is that if it hadn't been mentioned that there were multiple eyes on it, which blinked occasionally, I would never have noticed anything’ (Q5)*.

## DISCUSSION AND CONCLUSION

Understanding patients' perspectives on AI‐assisted colonoscopy requires acknowledging that they initially focus on the discomfort of the procedure, the related distress and feeling of vulnerability.

Our results showed that interpersonal factors are central during colonoscopies, with participants highlighting the inability of AI to provide empathy as a key limitation. While consistent with literature underscoring the importance of clinician empathy in patient acceptance [22], our findings demonstrate that these perceptions also apply when AI is visibly integrated in real‐time during the procedure. Increased procedural discomfort associated with insensitive physician behaviour has been linked to decreased compliance with future procedures [23]. Patients generally favour clinicians over AI, although they acknowledge AI as a supplement in healthcare [24]. Accordingly, compliance appears lower when AI replaces rather than supports the clinician [25], whereas empathy during the procedure fosters trust and confidence [26, 27].

Generally, accuracy seems to be perceived as one of the greatest strengths of AI in healthcare, but also one of the greatest potential weaknesses by patients, depending on how well the clinician's expectation matches the system's accuracy. Patients have expressed concerns about unfamiliar and novel situations where the accuracy may be lower than expected, increasing the risk of undetected errors. The potential deskilling of clinicians over time due to dependence on new technology has also been reported as a patient concern [6]. This was also reflected in our study, where AI‐assisted colonoscopy was perceived as something positive, but requiring clinicians to understand and respect its limitations and risks. In a previous survey of patients referred to colonoscopy, 65.2% reported that they would prefer their clinician to use AI‐assistance, but traditional indicators of endoscopist competence such as adenoma detection rate (ADR) were valued more importantly for the patients [28]. Participants' responses in our study suggest an implicit distinction between AI as a supportive tool and as an autonomous actor, particularly in relation to perceived responsibility for diagnostic errors and decision‐making.

Safety and quality concerns may create barriers between patients and the healthcare system during implementation of AI [29]. Addressing concerns, including the risk of misdiagnosis, AI safety, data security, bias in data sources, privacy breaches, reduced time with clinicians, and higher costs is important [9, 10, 30]. While our participants expressed these concerns, they were not pronounced. According to our findings, patients' acceptance of AI‐assistance does not appear to constitute a major obstacle provided that the clinician remains responsible for the procedure and the clinical decisions.

Successful implementation will depend not only on the clinical performance of the system, but also on transparent communication with patients regarding its role in the procedure. A key finding was that most patients underlined the importance of short and concise information. The degree and content of information that patients receive about AI‐assistance can be an important factor in how well they accept it [30]. However, information must be personalized and adapted to the patient's situation [26]. This variability was illustrated in our study, where participants differed in both the amount and type of information they wanted before and during the procedure.

It is also important to acknowledge the limitation of providing critical oral information while sedative medication is still active. Many participants in our study did not recall being informed about AI before or during the procedure, despite information being provided every time. All patients with absent recall had received midazolam, a sedative known to impair memory formation.

Furthermore, colonoscopy is a procedure often accompanied by concerns about diagnosis and potential discomfort. In this context, patients tend to focus primarily on their own body and the procedure itself, which may reduce their attention to external elements such as AI systems.

Our study has several strengths and limitations. PPI ensured participants felt well informed when deciding whether to participate. Although interviews were conducted days after the procedure and thus not influenced by residual sedative effects, sedation may have affected patients' memory encoding during colonoscopy. The narrow, well‐defined aim enabled a smaller sample size and more in‐depth analysis. The use of specific theories to guide planning and analysis provided a structured framework supporting the smaller sample size [31]. Transcript‐based analysis also reduced the risk of errors compared with memory‐ or note‐based analysis [18]. Inclusion criteria requiring ability to speak Danish, hospital attendance and interview participation may have introduced selection bias by excluding groups such as older and socioeconomically disadvantaged individuals, who are already underrepresented in the literature [6]. This limitation may affect the applicability of our results to the broader population. Future studies could include individual interviews with patients unable to attend group sessions due to language, physical, mental or cognitive barriers, potentially providing less biased insights. Furthermore, the exclusion of patients with suspected or confirmed colorectal cancer identified during the procedure may have reduced representation of more emotionally complex colonoscopy experiences, potentially limiting the generalizability of our findings to this subgroup. The present study was not designed to explore broader conceptual aspects of AI, such as the distinction between AI as a supportive tool and AI as a more autonomous actor. Future studies should investigate how patients perceive these different roles of AI and how such perceptions may influence acceptance and trust in AI‐assisted healthcare.

In conclusion, AI cannot replace clinicians in vulnerable situations such as during a colonoscopy. Empathy remains essential for patients, particularly because of procedural discomfort, associated distress and sense of vulnerability. AI‐assisted colonoscopy does not appear to affect patients' trust in clinicians, rather their trust in clinicians shapes their trust in AI. Information on AI‐assistance should be concise, brief and adequate to remain relevant for patients.

## AUTHOR CONTRIBUTIONS

**Marianne Krogsgaard:** Methodology; validation; supervision; formal analysis; investigation. **Ronja M. B. Lagström:** Conceptualization; investigation; writing – original draft; visualization; writing – review and editing; formal analysis; project administration; data curation; methodology. **Bahara Besmalahi:** Investigation. **Astrid Kindt:** Investigation. **Mustafa Bulut:** Conceptualization; methodology; resources; supervision.

## FUNDING INFORMATION

The authors have nothing to report. The study was conducted as part of the first author's PhD research.

## CONFLICT OF INTEREST STATEMENT

The authors declare no conflicts of interest.

## ETHICS STATEMENT

Participation was voluntary, and all patients provided written informed consent. In accordance with the Danish Health Care Act § 46 (5), the study was approved by the Head of the Department at Zealand University Hospital and registered in the Region Zealand record of processing activities (p‐2024‐16974).

## Supporting information


**Data S1:** Consolidated criteria for reporting qualitative research (COREQ) checklist (1).

## Data Availability

The data that support the findings of this study are available on request from the corresponding author. The data are not publicly available due to privacy or ethical restrictions.
