# Supplementary material for: Patients' experiences and attitudes toward the use of real‐time artificial intelligence‐assisted colonoscopy
Source: Colorectal Dis. 2026 Aug 7;28(8):e70574. doi: 10.1111/codi.70574 (PMC13451613; doi:10.1111/codi.70574)
Supplement: Supplementary file 1 [file CODI-28-0-s001.docx]

**Consolidated Criteria for Reporting Qualitative Research (COREQ) checklist** (1)

**Domain 1: Research team and reflexivity**

**Personal Characteristics**

1. Interviewer/facilitator: The first author (RL) conducted all interviews together with one of the co-authors (MK, AK or BB).

2. Credentials: The researchers´ credentials are MD or PhD.

3. Occupation: The researcher´s (RL) occupation at the time of the study was MD and PhD student.

4. Gender: The researcher is female (not stated, but her name is female).

5. Experience and training: The researcher (RL) is an MD, supervised by an experienced qualitative investigator and senior researcher (MK).

**Relationship with participants**

6. Relationship established: The primary investigator (RL) was responsible for the recruitment and talked to all patients prior to the procedure and the interview.

7. Participant knowledge of the interviewer: The participants knew about the interviewer’s occupations, and the aim of the study, and that it was a part of a PhD project. This is not described in the manuscript.

8. Interviewer characteristics: No characteristics, except occupation, were reported about the interviewer/facilitator.

**Domain 2: study design**

**Theoretical framework**

9. Methodological orientation and

Theory: Thematic analysis was used to analyze data. A semantic approach was used.

**Participant selection**

10. Sampling: Purposive sampling was used.

11. Method of approach: Participants were approached face-to-face and by phone.

12. Sample size: 20 patients

13. Non-participation: 3 patients did not wish to be invited. 25 patients did not wish to participate/could not participate (due to transport/other plans). 9 patients cancelled. Reasons are not reported in detail.

**Setting**

14. Setting of data collection: Data was collected at the same hospital as the initial procedures were performed.

15. Presence of non-participants: Only participants and researchers were present.

16. Description of sample: The sample is described in Table 1.

**Data collection**

17. Interview guide: An interview guide with questions and prompts was developed by the authors prior to the focus groups.

18. Repeat interviews: Six interviews were carried out.

19. Audio/visual recording: The researcher did use audio recording to collect the data.

20. Field notes: Field notes were made during and after each interview. This is not reported in the manuscript (due to the word limitation).

21. Duration: The duration of the interviews was 1-2 hours. This is not reported in the manuscript (due to the word limitation).

22. Data saturation: Data saturation is mentioned in the manuscript. This was also discussed in the research group throughout the project.

23. Transcripts returned: Transcripts were not returned to participants for comments and/or corrections. This is not reported in the manuscript.

**Domain 3: analysis and findings**

**Data analysis**

24. Number of data coders: All transcripts were coded by the first author (RL) and validated by one experienced qualitative investigator (MK).

25. Description of the coding tree: We did not provide a description of the coding tree, but it is available (in Danish) on request.

26. Derivation of themes: Themes were derived from the data.

27. Software: The software NVivo 15 was used to manage the data. This is not reported in the manuscript (due to the word limitation).

28. Participant checking: Participants did not provide feedback on the themes.

**Reporting**

29. Quotations presented: Participant quotations are presented to illustrate the themes. They are identified with participant numbers.

30. Data and findings consistent: There is consistency between the data presented and the findings.

31. Clarity of major themes: Major themes are clearly presented in the findings.

32. Clarity of minor themes: There are descriptions of diverse cases to illustrate the variation, as part of the major themes.

**Reference**

1.	Tong A, Sainsbury P, Craig J. Consolidated criteria for reporting qualitative research (COREQ): a 32-item checklist for interviews and focus groups. Int J Qual Heal care J Int Soc Qual Heal Care. 2007 Dec;19(6):349–57.
